# Correspondence

**Published:** 1887-01

**Authors:** G. R. Thomas

**Affiliations:** Pasadenca, Cal.


### CORRESPONDENCE.

PASADENCA, CAL., Dec. 17, 1886.

MY DEAR DR. TAFT:—In looking over the proceedings of the Ohio State Dental Society in the December number of the REGISTER, which I peruse with as much pleasure and interest as ever, although I am no longer tied to a dental chair, I observe a statement from you giving your first impressions of Dr. Younger's implantation operation, and, although I have had quite a little experience in transplantation, as you know, I could fully sympathize with your feelings of incredulity until last week, when, on a flying business trip to San Francisco, I snatched just time enough to call upon Dr. Cummings, the subject of said operation, to see for myself the result and appearance at the present time, and, to my astonishment and gratification, I found a presentation of as perfect adaptation as any tooth in his mouth. It is a left superior second bicuspid. I tested it most thoroughly in every way, reaching up under the gum margin as far as the process would allow. As yet there is no evidence of absorption or irritation, and the only discoverable difference between it and its neighbors, is upon percussion, the same solid, rock-like condition is met with as in replanted teeth. The color, occlusion, and physiological conditions are perfect.

The tooth, which this one replaces, Dr. Cummings tells me, was removed twelve years prior to this operation, and the implanted tooth had been removed three weeks prior to the latter, creates no surprise, but the former opens up a field of operation, the possibilities of which we *can not, we dare not* predict. Of course the time, as yet, is too short to be able to establish the utility of the operation, but, as Dr. Keely says, "a replanted tooth has done good service for thirty years," still, there are those who decry the operation of replanting, because a large percentage of cases fail sooner than artificial dentures and bridge work, and yet they are regarded as practical. If Dr. Younger's mode of operating proves as successful as it bids fair to, prosthetic dentistry, which is carrying on its shoulders Messrs. Bridge,

Crown, Pivot & Co., may as well drop the burden and throw up the sponge.

I did not have time to call upon Dr. Younger and learn his ideas of the practicability of the operation, but his work gives evidence of a brain behind it of heroic tendencies, and his operations will be watched with more than ordinary interest.

I observe that Uncle Jerry says that this climate is decidedly favorable to this class of operations. Well, I don't know but he is right, for I can testify to the fact that this climate is favorable to health, happiness, and comfort as no other is that I know any thing about, and, although old Detroit is as beautiful a city as this continent can boast of, I have no further use for her climate, and I judge, from the number of Eastern people who are constantly coming here, that I am not alone in this feeling, while we look out upon the nearest snow line forty miles distant, we are basking in the sunshine of perpetual summer, surrounded by all the fruits and flowers that such conditions foster.

I am most sincerely and truly yours,

G. R. THOMAS.

